# Editorial: Case reports in forensic psychiatry 2024

**DOI:** 10.3389/fpsyt.2025.1596975

**Published:** 2025-04-08

**Authors:** Sara Sablone, Giovanna Parmigiani, Anna Margari

**Affiliations:** ^1^ Section of Legal Medicine, Interdisciplinary Department of Medicine, Policlinico-University of Bari “Aldo Moro”, Bari, Italy; ^2^ Department of Law, University of Modena and Reggio Emilia, Modena, Italy; ^3^ Section of Criminology and Forensic Psychiatry, Interdisciplinary Department of Medicine, University of Bari “Aldo Moro”, Bari, Italy

**Keywords:** forensic psychiatry, case reports, case studies, forensic medicine, mental illness

Case reports serve multiple crucial purposes in forensic and legal medicine, forming the basis for new hypotheses, documenting novel observations, describing rare conditions, and offering significant educational value. By focusing on methodology, scientific investigations require sample sizes tailored to the specific research objective, with the choice of methods dictating the necessary sample size. At one extreme of the methodological spectrum, large-scale investigations such as genome-wide association studies (GWAS) require substantial samples to detect tiny genetic effects. Conversely, at the other extreme, case reports can yield significant insights even with a single-subject design, thereby underscoring the versatility of research methodologies in contributing to scientific knowledge. This approach is particularly true in forensic and legal medicine, where case reports offer in-depth analyses of individual cases, focusing on outliers. This granular approach allows for nuanced aspects’ exploration, thus revealing in singular cases the complex interplay of factors that may not be apparent in broader population studies.

In pursuit of outliers, we showcase the selected case reports published in this Research Topic. De Pieri and Suardi explore the impact of cultural biases and integration on sexual violence in novel socio-geographic contexts. They uniquely combine literature analysis with two case studies to examine how cultural factors influence power dynamics, perceptions of coercion, and consequences of sexual assault. The research highlights the need for integrating anthropological and ethnopsychiatric knowledge in forensic assessments and calls for early detection of non-acculturation elements to prevent criminal behaviors.

Lodde et al. underscore the rising need for forensic psychiatrists to understand online crime. A 27-year-old male, charged with image-based sexual abuse, was diagnosed with Unspecified Personality Disorder with narcissistic traits. The assessment, complicated by symptom exaggeration, highlights the importance of contextual knowledge of internet dynamics for accurate evaluations in the digital era.

Smith et al. contributed by reporting the case of a geriatric male patient with no previous history of delinquency who committed multiple aggressive acts and stalking offenses in his later life. During the forensic-psychiatric assessment (highlighting persistent delusional disorder based on pronounced symptoms and rigid personality traits), the patient declined neuroimaging scans but then consented to them. The doctors found a glioblastoma, an organic brain disorder (OBD), located in the brain areas crucial for executive function, emotional control, and social cognition, thus providing valuable insights into his behavioral changes and the new-onset delinquency. The multifaceted pitfalls and challenges of OBDs in forensic-psychiatric settings are pointed out in this case, which emphasizes the need for greater awareness and sensitivity towards these conditions, especially in elderly groups with externalized deviant behaviors.

Tomassini et al. reported three cases of drug overdose death resulting from a prison riot during the initial wave of the SARS-CoV-2 pandemic in Italy. Methadone was identified as the main cause of death in two cases after conducting a comprehensive toxicological analysis and exploring the synergistic effects of substances. The challenges of determining if these deaths are accidental, intentional (suicidal), or a result of uncontrollable drug consumption during a riot are highlighted. The authors underscore how mass intoxication and overdose may be the result of prison riots, which exacerbate drug abuse issues and pre-existing psychiatric disorders within this critical context.

Capasso et al. explored the importance of forensic medical assessment and its incorporation in psychodiagnostic examination to determine the correct nosographic classification for evaluating and quantifying biological damage. The reported case is about the resisting administration’s alleged failure to fulfill obligations from the contract and employment relationship, as well as violating safety regulations, which caused the workplace accident reported by an Air Force Sergeant. After the accident, the soldier claims to have experienced barotrauma, hearing loss, tinnitus, and reactive post-traumatic stress disorder as a result of the traumatic event. This case report emphasizes the critical role of integrated forensic medical and psychodiagnostic assessments in evaluating workplace accidents.

In conclusion, this Research Topic underscores the critical role of case reports in advancing forensic psychiatry. Through this outlier exploration, we provide handy information for more effective forensic practices and policies.

